# Extracorporeal photopheresis in stiff person syndrome

**DOI:** 10.3389/fimmu.2024.1519032

**Published:** 2024-12-16

**Authors:** Yandy Marx Castillo-Aleman, Pierre Christophe Krystkowiak

**Affiliations:** ^1^ Department of Immunology, Abu Dhabi Stem Cells Center (ADSCC), Abu Dhabi, United Arab Emirates; ^2^ Department of Neurology, Specialized Rehabilitation Hospital/Capital Health, Abu Dhabi, United Arab Emirates

**Keywords:** extracorporeal photopheresis, immunomodulation, movement disorders, neuroimmunomodulation, stiff person syndrome

## 1 Introduction

Stiff person spectrum disorders (SPSDs) are a rare group of neuroimmunological disorders characterized by progressive rigidity and triggered painful spasms of the limb muscles. Despite the first description by Moersch and Woltman in 1956 of the formerly coined “stiff man syndrome” (1) or as a gender-neutral term of “stiff person syndrome (SPS),” (2) this condition has a clinical spectrum that includes not only classical SPS but also other SPS variants, such as progressive encephalomyelitis with rigidity and myoclonus (PERM) (3).

Classical SPS is the predominant clinical form and presents as an insidious onset with rigidity and stiffness of the trunk muscles, which advance to joint deformities, impaired posturing, and abnormal gait (1, 3). Patients may also develop painful generalized muscle spasms triggered by unexpected stimuli and may be associated with other autoimmune disorders (3, 4). The clinical features of SPS variants include focal or segmental SPS (“stiff limb syndrome”), jerky SPS, SPS with epilepsy, SPS with dystonia, cerebellar, and paraneoplastic variants (3–5).

In addition to axial and limb muscle stiffness and diffuse myoclonus, patients with PERM (“SPS-plus syndrome”) exhibit relapsing–remitting brain stem symptoms, breathing issues, and prominent autonomic dysfunction (6).

Despite significant advances in the treatment of SPSDs, the prognosis remains unpredictable, with an inadequate response in many patients, leading to severe disability and sudden death (5, 7). Moreover, most patients receiving standard-of-care medications may require progressively higher doses, leading to intolerable adverse events (5), among other limitations of pharmacological interventions discussed later. Therefore, there is a need to identify innovative therapies in which we describe the potential use of extracorporeal photopheresis (ECP) as a rational approach for patients with SPSDs, specifically classical SPS. Of note, there are no case reports, patient cohorts, or clinical trials have been reported on the use of ECP in SPS yet. Accordingly, this study aims to propose ECP as a potential treatment for SPS by analyzing the current evidence supporting its clinical application.

## 2 Etiopathophysiology

SPSDs are associated with high titers of autoantibodies to different antigens of inhibitory synapses, generating low level of synthesis and release of γ-aminobutyric acid (GABA) on presynaptic or postsynaptic neuronal junctions within the central nervous system (CNS), resulting in impaired functioning (3, 8).

Glutamic acid decarboxylase (GAD), a cytoplasmic enzyme with two isoforms (GAD67 and GAD65) that transforms glutamate into GABA, has been widely recognized as a primary target identified in classical SPS, predominately anti-GAD65 antibodies (3, 8).

However, other autoantibodies have also been reported, and various correlations with SPSD variants have been established, including antibodies against GABA receptor-associated protein and dipeptidyl-peptidase-like protein-6 (DPPX) in classical SPS, amphiphysin and gephyrin in paraneoplastic variants, and glycine receptor associated with PERM (3, 9).

The classical SPS etiopathophysiology has been explained by the B cell-mediated inhibition of GABAergic neurons and their synapses, whereas GAD65-specific T cells accumulated in the CNS could drive the intrathecal GAD65 IgG production (3, 10). T cell-mediated cytotoxicity has also been reported in SPS, as GAD65-specific T cells can initiate cytotoxic immune responses (11).

Despite evidence suggesting that GAD65-specific T cells are likely to be scarce and mainly confined to the naïve repertoire in blood (10), there is a systemic and oligoclonal immune response mediated by stable B cell clones (12) leading to serum titers that are 50-fold higher than cerebrospinal fluid (CSF) titers (4).

Interestingly, the serum and CSF anti-GAD antibodies first reported by Solimena et al. in a patient with SPS, diabetes mellitus, and epilepsy (13) were not consistently correlated with the clinical fluctuations of the disease (4, 11). These autoantibodies are directed to GAD65 intracellular antigens and have been postulated to interact with peptide fragments during GABA exocytosis on neuronal surfaces, exerting a change in the synaptic transmission by blocking either GAD function or synthesis (14).

GAD65-specific memory T cells could enter the CNS and mount effector responses against GAD65-expressing neurons, including infiltrating CD8^+^ T cells (11) detected in the spinal cord of deceased patients with SPS, along with neuronal loss and axonal swelling (15).

## 3 Current therapies

SPS treatment includes drugs that increase the GABAergic tone in combination with immunomodulating or immunosuppressant agents (4, 5).

At the onset of SPS symptoms or appropriate diagnosis, diazepam or other benzodiazepines (GABA agonists) are commonly used as the cornerstone of symptomatic therapies. However, other drugs, including muscle relaxants, botulinum toxin injections, and centrally acting agents, are also used (11).

SPS immunotherapies are usually the first-line treatment and include corticosteroids, therapeutic plasma exchange, high-dose intravenous immunoglobulins (IVIg), and subcutaneous immunoglobulins (SCIg) (11).

Anti-B cell therapies have recently been proposed as a rational approach in second-line therapies, along with mycophenolate mofetil, azathioprine, or a combination of therapies (4, 5, 11). Treatment with autologous anti-CD19 chimeric antigen receptor (CAR) T cells has also been successfully reported in a patient with refractory SPS (16). Third-line therapies include cyclophosphamide or a combination of therapies (e.g., IVIg and rituximab or mycophenolate mofetil) (11).

Autologous non-myeloablative hematopoietic stem cell transplantation (HSCT) in disabled patients with SPS has also been reported, despite its variable beneficial effects (fourth-line therapies) (11, 17).

Commonly, SPS pharmacological treatment is combined with nonpharmacological interventions (e.g., selective physical therapy, deep tissue massage techniques, heat therapy, osteopathic and chiropractic manipulation, and acupuncture) in a multifaceted approach (11). Nevertheless, current pharmacological interventions lead to heterogeneous clinical responses and pose various limitations (**Table 1**), which support exploring further strategies, such as ECP, that might be added to the SPS therapeutic armamentarium.

**Table 1:** Potential limitations of SPS pharmacological interventions (4, 5, 9, 11, 15).

Category	Examples	Potential limitations
Symptomatic therapy	- Benzodiazepines - Baclofen - Botulinum toxin	- Heterogeneity of clinical outcomes - Treatment response per phenotype - Differences in individual tolerability - Adverse events (e.g., respiratory depression) - Symptomatic withdrawal or rebound after treatment discontinuation (e.g., if malfunctioning baclofen pumps) - Combination of symptomatic therapies often required
First-line immunotherapy	- Corticosteroids - IVIg/SCIg - Plasma exchange	- Adverse events (e.g., increased risk of developing diabetes, infusion reactions, risk of thrombosis, renal dysfunction, stroke, aseptic meningitis) - Treatment response per phenotype - Long-term maintenance treatment is needed - Logistical and financial issues
Second-line immunotherapy	- Rituximab - Mycophenolate mofetil - Azathioprine	- Heterogeneity of clinical outcomes with subtherapeutic responses - Long-term maintenance treatment is needed - Adverse events (e.g., severe immunosuppression)
Third-line immunotherapy	- Cyclophosphamide - Combination of therapies	- Heterogeneity of clinical outcomes with subtherapeutic responses - Predictors of response are poorly defined - Adverse events (e.g., severe immunosuppression)
Fourth-line immunotherapy	- CAR-T cells - Autologous HSCT	- Heterogeneity of clinical outcomes with subtherapeutic responses - Predictors of response are poorly defined - Logistical and financial issues

IVIg/SCIg, intravenous/subcutaneous immunoglobulins; CAR-T, chimeric antigen receptor (CAR) T cells; HSCT, hematopoietic stem cell transplantation.

## 4 Rationale supporting ECP

ECP is a leukapheresis-based immunotherapy in which autologous leukocytes are exposed to a photosensitizing agent and ultraviolet-A (UVA) irradiation before being reinfused. The photosensitizing agent 8-methoxypsoralen (8-MOP) conjugates with the DNA of leukocytes upon UVA photoactivation, resulting in the inhibition of DNA synthesis and cell division and the induction of apoptosis, generating a cascade of events (18).

It has been approved for the palliative treatment of cutaneous T cell lymphoma, and many other indications have been successfully explored, including graft-versus-host disease, rejection of solid organ transplantation, and a few autoimmune diseases (18).

During a regular ECP procedure, nearly 5%–10% of the total blood-circulating mononuclear cells are drawn and exposed to 8-MOP and UVA, and the susceptibility to ECP-induced apoptosis varies from cell to cell (18, 19). For instance, B and T cells are highly susceptible to 8-MOP/UVA exposure, whereas monocytes and regulatory T cells (Tregs) are more resistant to ECP (18).

ECP exerts “direct effects,” including apoptosis of treated leukocytes, followed by phagocytosis, which trigger cascades of downstream “indirect effects.” (20) Many cell interactions initiate a cascade of immunological changes, differentiation of monocytes into dendritic cells (DCs), and successive presentation of antigens (18). ECP-treated cells also recruit other modulators, such as phagocytes, via soluble and membrane-bound “find me” signals (21). The “indirect effects” of ECP include the eradication of (pathogenic) clonal cells, a shift in antigen-presenting cell (APC) populations, changes in cytokine secretion, and modulation of Tregs and regulatory B cells (Bregs) (20, 22).

### 4.1 Blood–brain barrier: An objection for ECP?

Although the CNS has been considered an immunoprivileged site, current evidence shows the effective recruitment of immune cells across the blood–brain barrier (BBB) into perivascular and parenchymal spaces (23).

T cell responses targeting CNS antigens are initiated in secondary lymphoid organs, and not in the CNS (10). In fact, activated T cells may penetrate the BBB, regardless of their specificity, and intrathecally are retained those T cells which encounter their cognate antigen (24).

In this regard, Skorstad et al. indicated that GAD65-specific T cells may first be activated in the periphery and later accumulate in the CNS, including proliferation and promotion of B cell differentiation into GAD65 IgG-producing plasma cells within the intrathecal compartment of patients with SPS (10).

Compared with serum anti-GAD65 antibodies, the CSF antibodies of patients with SPS exhibit a 10-fold higher binding avidity, indicating intrathecal synthesis by clonally restricted GAD65-specific B cells driven by local antigens within the confines of the BBB (4, 10).

Additionally, DCs involved in both primary and secondary immune responses can migrate not only into the perivascular space under degeneration and neuroinflammation (23), but also into the CSF-drained spaces of the CNS, even in the absence of neuroinflammation (25, 26). Furthermore, DCs can traffic to peripheral lymphoid organs (e.g., cervical lymph nodes) and present CNS antigens to T cells in the periphery (26).

Therefore, although the BBB may diminish the effects of ECP, the periphery–CNS trafficking of immune cells and anti-GAD65 antibody production can justify its investigational use in preclinical models and, eventually, in clinical trials.

### 4.2 Weighing the ECP pros and cons

Unlike standard immunosuppressive therapies, ECP does not cause general immunosuppression; instead, it appears to exert complex specific effects (27) across different immune pathways (22).

Analyzing the various immune specificities in the variations of the clinical phenotypes of SPSDs, we herein describe some potential mechanisms and caveats of ECP to be considered in the context of classical SPS.

#### 4.2.1 Arguments in favor of ECP feasibility for SPS

##### 4.2.1.1 Postulated ECP mechanisms in classical SPS

– Considering the pathophysiology of SPS and the presence of GAD65-specific T cells in the CNS that drive intrathecal production of GAD65 IgG (10), the indirect effects of ECP may be preponderant in SPS.
– ECP induces apoptosis that first appears in activated lymphocytes (e.g., affinity-maturated B cell clones), which are more sensitive to apoptosis than other cells (28).
– Despite the intrathecal synthesis of GAD65 antibodies indicating that T cells from CSF cells can be more relevant than those from blood in SPS (9), there is also persistent systemic oligoclonal production of GAD65-specific IgG (12).
– Apoptotic GAD65-specific B and T cells may be phagocytosed by DCs that present antigens to T-helper (CD4^+^) cells, consequently raising specific tolerance to the clonal T cell population (29).
– ECP increases CD4^+^ CD25^+^ Foxp3^+^ Tregs induced by a tolerogenic phenotype of DCs in contact with apoptotic cells in the periphery (23, 30, 31), which can also gain access to the CNS during neuroinflammatory autoimmunity events (23).
– Activated T cells of any specificity can penetrate the BBB; however, only those reactivated in the CNS are intrathecally retained (10).
– Following the increase of Tregs induced by ECP, the CSF-drained spaces of the CNS are eventually accessed by such activated cells, even in a non-neuroinflammatory environment (25).
– Before the blood-derived leukocytes enter the CSF, they first pass-through fenestrated capillaries and accumulate in the choroid plexus parenchyma, in which resident DCs can skew immune cells (25). ECP-induced apoptotic (GAD65-specific) cells can induce a tolerogenic phenotype of such unique DCs.
– Blood-borne cytokines that cross the BBB and enter the CSF and interstitial fluid spaces of the CNS may also favor this immune regulation (30).
– Circulant GAD65-specific T and B cells may undergo immunogenic cell death and serve as the major sources of subsequent GAD65 antigen processing and presentation (32).
– Similarly, other SPS immune-modulating therapies can tackle the peripheral compartment (e.g., therapeutic plasma exchange (33), high-dose IVIg (34), B cell depletion (16, 35), and autologous non-myeloablative HSCT (17)).
– Substantial Th2 cytokine levels drive a T cell–B cell collaboration and may drive intrathecal production of oligoclonal IgG in SPS (10). In this regard, ECP may restore the Th1/Th2 balance and induce tolerance (19).
– Furthermore, ECP can decrease the pro-inflammatory T cell subset of Th17 cells that commonly cross epithelial blood–CSF barriers (19).

##### 4.2.1.2 Additional elements to be considered

– Adequate safety profile of ECP (36).
– Different immune specificities may exist within the same patient with SPS (11), and he/she may still benefit from ECP.
– The coexistence of nuclear and cytoplasmic autoantibodies that reflect immune responses to multiple CNS and other tissue-specific antigens (4) would also be addressed by ECP.
– The clonal pattern of GAD65 antibodies in the CSF remains stable for several years (9).
– ECP may be feasible in the case of certain concomitant autoimmune diseases with SPS (e.g., type 1 diabetes mellitus, ClinicalTrials.gov identifier NCT05413005, an ongoing clinical trial in our center) (37).
– Previous and ongoing ECP-based clinical trials on other immune-mediated CNS disorders (e.g., multiple sclerosis –MS, NCT05168384, which is also active in our hospital) (38).

Previous clinical experience with ECP has been documented in other immune-mediated CNS disorders, such as MS, in which a few case reports and small clinical trials verified the safety of ECP, but the results were inconclusive in terms of efficacy (39, 40). For instance, Besnier et al. reported that ECP transiently modified the course of severe secondary chronic progressive MS with a rebound after treatment discontinuation (41), and Cavaletti et al. reported evidence of adequate efficacy in a subgroup of patients with MS not responsive to or ineligible for standard immunomodulating treatments (42).

Regarding the use of photopheresis in patients with classical SPS, our group has proposed to execute the termed OPTION study, a pilot open-label trial using ECP as an add-on investigational intervention (NCT06703333) comprised of one ECP cycle (two consecutive days) every other week for three months, followed by one ECP cycle every month for additional three months. This trial will evaluate safety outcomes as the primary endpoints, but the efficacy will be preliminarily assessed through changes in the Distribution of Stiffness Index (DSI) and Heightened Sensitivity Score (HSS) (43).

**Figure 1:**
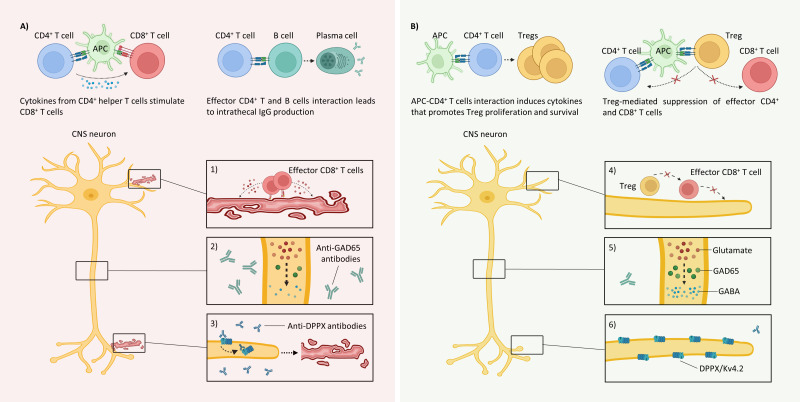
Immunopathology of classical SPS and the postulated mechanisms of ECP **(A)** Various peripherally circulating cells recognizing GAD65 peptides can traffic to the CNS and mount immune responses, leading to dysfunctional synapses because of the following: *1) CD8^+^ T cell-mediated cytotoxicity:* The GAD65 epitopes presented in the MHC-I molecules recognized by autoreactive T cells initiate cytotoxic immune responses by releasing perforin and granzyme **(B)** *2) Loss of inhibitory signals:* Although neurons do not internalize GAD65 antibodies, they recognize linear epitopes and limit GABA synthesis. *3) Antibody-mediated neuronal hyperexcitability and cytotoxicity:* Anti-DPPX antibodies initiate the internalization of the accessory proteins DPPX and KV4.2 (left), which produce hyperexcitability and cytotoxicity (right). *B)* The following postulated mechanisms of ECP may result in homeostasis in classical SPS: *4) Tolerance to GAD65-expressing neurons: Treg*-mediated suppression of effector GAD65-specific CD4^+^ and CD8^+^ T cells. *5) Inhibitory signal restoration:* Decreases in intrathecal GAD65 IgG production may regulate inhibitory interneurons. *6) Membrane surface stabilization:* The decrease in anti-DPPX antibodies reduces the internalization of both DPPX and Kv4.2 and stabilizes neuron membranes. APC, antigen-presenting cell; DPPX, dipeptidyl-peptidase-like protein-6; GABA, γ-aminobutyric acid; MHC-I, major histocompatibility complex class I. Created with BioRender.com.

**Figures 1A, B** summarize the main etiopathophysiological CNS events and postulated mechanisms of ECP in SPS, respectively.

#### 4.2.2 Caveats and limitations of ECP feasibility in SPS

– The peripheral blood (main ECP direct target) is separated from the diseased organ by the BBB.
– GAD65-specific T cells are likely to be scarce in peripheral blood, and the intrathecal synthesis of GAD65 antibodies indicates that CSF T cells can be more relevant than blood T cells (9).
– Serological markers are not commonly correlated with clinical severity, and the systemic synthesis of GAD65 antibodies may be insufficient for mediating SPS (44, 45).
– Different epitope specificity between serum and the CSF may reduce the potential efficacy of ECP (9).
– ECP may be a rational approach for certain disorders of the spectrum (e.g., classical SPS) but it is not feasible for all SPSDs (e.g., paraneoplastic variants).
– Lack of controlled clinical trials due to the low prevalence of SPS.
– Potential ECP-induced immune regulation may not be clinically relevant.
– The availability of ECP providers may also be challenging, including logistical and financial issues, vascular access needs (which, in the case of poor peripheral venous accesses, may eventually require the insertion of a central venous catheter), potential adverse events, and the uncertain ECP schedule and duration, which ultimately depend on the treatment response.

With the aforementioned pieces of evidence, being a well-tolerated and safe procedure with long-term effects in approved indications, ECP might overcome various gaps faced with current SPS treatments, which commonly provide a shorter duration of clinical improvement or variable beneficial effects (5, 7, 16, 17). For instance, instead of the therapeutic approach of controlling disease symptoms (e.g., benzodiazepines and muscle relaxants), targeting some of the critical cells involved in the etiopathophysiology (e.g., anti-B cell therapies) or even “rebooting” the immune system (autologous HSCT), ECP possesses established immunologic effects that, in combination with those treatments, may gradually modulate the dysregulated immune response observed in SPS.

## 5 Conclusions

Although the exact mechanism of action of ECP remains unclear and requires further studies in SPS, its wide-ranging immunomodulatory effects may be beneficial in this disabling disorder. By exploring the effect of ECP in preclinical models and formal clinical trials, this approach may also foster its use in SPS and potentially in other neuroimmunological diseases.
